# A Study of Trench Feet

**Published:** 1918-02

**Authors:** 


***A Study of Trench Feet.*** By M. Victor Raymond, Médecin-Major de 1re classe. Trans. and Abs. from the *Archives de Médecine et de Pharmacie Militaires*, November, 1915, t. LXIV, N° 5, p. 475.

R. describes the symptoms of trench feet under the following heads : sensory, motor, trophic, and general disturbances.

*Sensory Disturbances.* Spontaneous, recurring *pains*, sometimes so violent as to interfere with walking and prevent sleep. In extreme cases it is necessary to inject morphine. The pains are felt chiefly in the front part of the foot and in the toes. They sometimes extend to the heel or even to the leg, the thigh, and the groin.

Sensitiveness to the touch is noticed chiefly on the sole of the foot at the heads of the metatarsi, extending thence towards the heel. Less frequently pain is felt if the metatarsal region is gripped and pressed from the sides of the foot. Occasionally pressure on the dorsum of the foot is painful; rarely, pressure behind the ankle. Less frequently there is sensitiveness along the inner face of the tibia and at the head of the fibula.

*Paresthesia* is even more common than the pains. The patient complains of a prickly sensation in the front part of the foot and in the toes. Burning sensations and cramps may be felt. One man said that his toe felt as if it were sticking through a hole in his shoe. Sometimes the numbness is complete. Besides, the toes are usually completely anesthetic, even to pricking. Although the anesthesia is generally confined to the toes, it may sometimes extend on to the dorsum of the foot.

*Motor Disturbances.* These are much rarer, and often result from the sensory disturbances. The author states that he has never observed paralysis or paresia of the feet. Often, however, patients find it difficult to walk, even when there is no pain.

*Reflex Disturbances.* Generally the patellar reflexes are normal. In some cases they have appeared slightly exaggerated; but in a few cases sub-normal. The author has always found the plantar reflex normal.

*Trophic Disturbances.* In order of frequency these are : edema, vaso-motor disturbances, blisters, and scabs.

*Edema and Vaso-motor Disturbances.* Swelling on the dorsal surface of the foot is frequent, but it rarely extends further. In some cases it is confined to the toes. The skin over the swelling is sometimes a violet red and may present a pseudo-phlegmonous appearance, accompanied by local hyperthermia.

In other cases the edema may be white and hard; or this appearance may exist without the edema.

In almost all the cases examined the Dorsalis pedis artery appeared normal, and there was no apparent vascularization of the veins. There was no varix.

*Blisters* appear on the dorsum of the foot, especially on or near the toes. They are usually simple, containing an abundance of *serum.* They may, however, be hemorrhagic.

*Scabs* form in the majority of cases under the blisters, affecting the skin and subcutaneous tissues. Necrosis, however, may be much more extensive, often including the whole of the toes. Although the author was unable to follow the development of such cases, he noticed on one patient that two toes, which seemed perfectly black and doomed to elimination, regained their circulation and color after five or six days.

*General Disturbances.* Usually there is no rise in temperature and no digestive, pharyngeal, or pulmonary disorders. Albuminuria was ncted in three cases.

*Evolution.* Invariably the patients have been serving in the trenches. The first symptoms do not appear, as a rule, until the patient has left the trenches. The difficulty in walking is first noticed as the patient is on his way from the trenches to the cantonment. The edema appears, often suddenly, as the patient removes his shoes. The blisters also appear at this time. The pains are noticed the first or the second night of the rest period, and they increase each day. Anesthesia reaches its maximum immediately, but gradually diminishes as the pains increase. In simple cases the disease runs its course in from two to three weeks. The anesthesia is the most persistent symptom. Both feet are usually affected, although one is commonly worse than the other.

*Pathology.* The pathogenic origin of the disease appears to the author to be a peripheral *neuritis* of the nerve fibers of the foot, that is, of the terminal branches of the sciatic nerve. This explanation appears to him more satisfactory than one based upon the interruption of the circulation. Arterial lesions might cause necrosis and cold, but would not produce the characteristic phenomena of the edema and the sensory disturbances. Besides, the artery supplying the affected region beats normally. Interference with veins might produce the edema, but not the dry gangrene or the sensory disturbances. Neuritis, however, may satisfactorily explain all of the changes observed.

*Etiology.* This problem is very obscure. The disease is clearly connected with the conditions in the trenches, for only men in the trenches ever contract it, although others are equally exposed to out-of-doors conditions.

*Age* is an important factor. The young (from 20 to 30 years of age) are more frequently affected than the reservists and territorials.

*The duration of the exposure* may vary. Three or four days may suffice to produce the symptoms, but often patients have been exposed from 8 to 15 days before the symptoms appeared. In one case, the patient did not contract the disease during his first exposure of four days, but when he returned to the trenches after his relief, he was affected at the end of the second day. In this case it appeared that the first exposure predisposed the patient for the attack.

Cases are seldom reported singly, but in groups of from 10 to 20 immediately upon the arrival of a unit at its rest cantonment. It therefore appears that some common cause acts in a similar manner upon all who are not immune. The author proceeds to discuss the possible causes :

*Infection.* The fever that is sometimes reported and albuminuria, which the author has himself observed, might lead to the supposition that infection played some part. On the other hand, the infrequency of general symptoms, the localization of the disturbances in the feet, and the absence of neuritis in any of the trunks not directly affected, argue strongly against the hypothesis of infection.

*Intoxication* appears to the author very unlikely.

*Cold*, the author believes, is not an exclusive cause, or even the principal one. The first cases were noted in October, 1914, when it was not cold. Many of the patients declared that they had not felt cold at any time. The temperature rarely falls below freezing in the region where the troops are stationed, and unless the men have been actually in the trenches they are never affected.

Besides, the symptoms of genuine freezing are very different; in such cases there is necrosis and a line of demarcation, but no sensory disturbances or edema of the unaffected regions.

*Humidity.* Almost invariably those affected state that they have been in trenches that were damp or flooded with water. The author noted particularly a corps of recruits of the same age, some of whom were stationed in low swampy trenches and others in dry ones along a plateau. Cases of trench feet invariably came from the low swampy sections. The effect of humidity upon the peripheral nerve system and in rheumatic disorders is well known.

*Inactivity.* The men are usually inactive in the trenches, either standing or sitting. Thus the blood vessels supplying nerves may be *dilated* and the nerve fibers irritated.

*Compression* from the shoes might be a contributing factor, but, as a matter of fact, none of the patients had noticed any tightness of their shoes. The author frequently observed that the edema did not appear until some time after the shoes were removed.

Infection, cold, and especially humidity, inactivity, and compression, are the possible causes of the condition noticed in trench feet. They probably act in varying degrees and combinations in the etiology of the disease.

Authorities are agreed that in disturbances of the peripheral nerves, such as, sciatica, radial paralysis, and neuralgia, the causal factors may be a series of conditions similar to those that give rise to trench feet. The author, therefore, is inclined to consider this malady a form of *neuritis* brought on especially by humidity and favored by the age of the patient. It appears to be the result of physical or mechanical agents, but it is possible to conceive that infection similar to that of rheumatism may be the responsible cause. Possibly the peripheral neuritis reported by the Japanese in the trenches before Mukden may have been of a similar nature.

*Diagnosis.* — The condition of trench feet is easily recognizable. It differs from ordinary freezing, which most closely resembles it, in that the suffering from freezing is much more intense and is not accompanied by anesthesia. Rheumatism is more distinctly localized in segments of a member or in articulations.

*Prognosis.* — The lighter cases may be expected to recover completely in from 20 to 30 days. The more serious and complicated form may continue indefinitely.

*Complications.* — Infection with suppuration, lymphangitis, or adenitis, may be observed in the ulcerated or gangrenous regions.

It should also be noted that a condition of trench feet may predispose a patient to actual freezing. In two instances it was learned that after the men had suffered several days from trench feet a single night of unusual cold, was sufficient to produce actual freezing.

*Treatment.* — The shoes should be removed and the feet placed at rest, and surrounded with cotton. If there are no lesions of the skin, warm baths and massage with some greasy substance, may be beneficial. When there are blisters or scabs, a simple aseptic dressing suffices. It is not necessary to open the blisters, as they dry up spontaneously. Sometimes the pain is so acute that narcotics or morphine injections are necessary.

*Prophylaxis.* — 1. Every effort should be made to keep the trenches dry by heating, drainage, or pavement.

2. The shifts in the trenches, especially if these are known to be damp, should be made as often as possible consistent with military necessity.

3. Activity should be encouraged, and the men should be well nourished and provided with warm drinks.

4. They should be made to remove their shoes in the rest cantonments, and, if possible, to take warm foot baths and massage their feet with some greasy substance.

5. The men's shoes should be carefully greased during the periods in the trenches.

6. Special precaution should be taken in the case of young men.

7. Whenever a man complains of pains in the feet he should be examined at once for signs of anesthesia, which are an indication for immediate relief and treatment. A few days of rest without evacuation, generally suffice for the complete cure of an incipient case, and so weeks may be saved by prompt detection and treatment.

